# Early versus delayed interventions for necrotizing pancreatitis: A systematic review and meta‐analysis

**DOI:** 10.1002/deo2.171

**Published:** 2022-10-10

**Authors:** Yousuke Nakai, Hideyuki Shiomi, Tsuyoshi Hamada, Shogo Ota, Mamoru Takenaka, Takuji Iwashita, Tatsuya Sato, Tomotaka Saito, Atsuhiro Masuda, Saburo Matsubara, Keisuke Iwata, Tsuyoshi Mukai, Hiroyuki Isayama, Ichiro Yasuda, WONDERFUL study group in Japan

**Affiliations:** ^1^ Department of Gastroenterology Graduate School of Medicine The University of Tokyo Tokyo Japan; ^2^ Department of Endoscopy and Endoscopic Surgery The University of Tokyo Hospital Tokyo Japan; ^3^ Division of Gastroenterology and Hepatobiliary and Pancreatic Diseases Department of Internal Medicine Hyogo Medical University Hyogo Japan; ^4^ Department of Hepato‐Biliary‐Pancreatic Medicine The Cancer Institute Hospital of Japanese Foundation for Cancer Research Tokyo Japan; ^5^ Department of Gastroenterology and Hepatology Kindai University Faculty of Medicine Osaka Japan; ^6^ First Department of Internal Medicine Gifu University Hospital Gifu Japan; ^7^ Division of Gastroenterology Department of Internal Medicine Kobe University Graduate School of Medicine Hyogo Japan; ^8^ Department of Gastroenterology and Hepatology, Saitama Medical Center Saitama Medical University Saitama Japan; ^9^ Department of Gastroenterology Gifu Municipal Hospital Gifu Japan; ^10^ Department of Gastroenterological Endoscopy Kanazawa Medical University Ishikawa Japan; ^11^ Department of Gastroenterology Graduate School of Medicine Juntendo University Tokyo Japan; ^12^ Third Department of Internal Medicine University of Toyama Toyama Japan

**Keywords:** acute necrotizing pancreatitis, drainage, endoscopic ultrasound, necrosectomy, walled‐off necrosis

## Abstract

**Objectives:**

Interventions for necrotizing pancreatitis are generally postponed until 4 weeks after the onset of acute pancreatitis, but there remains controversy about whether we should always wait >4 weeks or can intervene early when necessary. This meta‐analysis was conducted to evaluate treatment outcomes of necrotizing pancreatitis according to the cut‐off defined in the revised Atlanta classification (≤4 vs. >4 weeks).

**Methods:**

Using PubMed, Web of Science, and the Cochrane database, we identified clinical studies published until March 2022 with data comparing outcomes of early and delayed interventions of necrotizing pancreatitis. We pooled data on adverse events, mortality, technical and clinical success rates, and needs for necrosectomy and open surgery, using the random‐effects model.

**Results:**

We identified 11 retrospective studies, including 775 patients with early interventions and 725 patients with delayed interventions. Patients with early interventions tended to be complicated by organ failure. The rate of adverse events was comparable (OR 1.41, 95% CI 0.66–3.01; *p* = 0.38) but the rate of mortality was significantly higher (OR 1.70, 95% CI 1.21–2.40; *p* < 0.01) in early interventions. Technical success rates were similarly high but clinical success rates tended to be low (OR 0.39, 95% CI 0.15–1.00; *p* = 0.05) in early interventions, though not statistically significant. Pooled ORs for necrosectomy and open surgery were 2.14 and 1.23, respectively.

**Conclusions:**

Early interventions for necrotizing pancreatitis were associated with higher mortality rates and did not reduce adverse events or improve clinical success. However, our results should be confirmed in prospective studies.

## INTRODUCTION

Acute pancreatitis (AP) is one of the most common gastrointestinal diseases,^1^ and about 20% of patients develop necrotizing pancreatitis.^2^ Pancreatic fluid collections (PFCs) are common local complications of AP, and the revised Atlanta classification^3^ categorized PFCs into the acute peripancreatic fluid collection, acute necrotic collection (ANC), pancreatic pseudocyst, and walled‐off necrosis (WON) depending on the time after the onset of AP (≤4 vs. >4 weeks) and the presence of necrosis. Since the presence of necrosis poses the patients with the risk of infection and sepsis‐related mortality, surgical debridement of necrotic tissue, even in the early phase of the disease, was historically considered beneficial in patients with necrotizing pancreatitis around the late 18th and the early 19th centuries.^4^ In those days, recovery from necrotizing pancreatitis was rare by non‐surgical management, but mortality after the surgical intervention was also reportedly as high as 50%.^5^ Since then, the paradigm shift has occurred from surgical interventions to the less invasive, step‐up endoscopic, or percutaneous approach.^6^ However, there remains controversy about whether we should always wait for >4 weeks from the onset of AP or intervene early when necessary.^7^, ^8^, ^9^ In previous studies,^10^, ^11^, ^12^ the timing of infection in ANC was sometimes earlier than 4 weeks from the onset of AP, and early interventions can potentially enhance the resolution of infected ANC if achieved safely. The debate on this timing of interventions has resurged since mortality and morbidity have decreased because of recent multidisciplinary non‐surgical management of necrotizing pancreatitis. The development of lumen‐apposing metal stents (LAMSs)^13^ or large bore metal stents^14^ for endoscopic ultrasound‐guided drainage made procedure time shorter with fewer adverse events.^15^, ^16^

We, therefore, conducted this meta‐analysis to evaluate treatment outcomes of early and delayed interventions for necrotizing pancreatitis, according to the revised Atlanta classification.

## MATERIALS AND METHODS

### Study overview

This systematic review and meta‐analysis aimed to evaluate treatment outcomes of early vs. delayed drainage of necrotizing pancreatitis and was conducted in accordance with the PRISMA (the Preferred Reporting Items for Systematic reviews and Meta‐Analyses) guideline.^17^ The protocol was registered in the database of UMIN (University Hospital Medical Information Network; registration number, UMIN000047225). This study was conducted by the WONDERFUL (WON anD pERipancreatic FlUid coLlection) study group, which consisted of expertized endoscopists, gastroenterologists, interventional radiologists, and epidemiologists at high‐volume centers in Japan (UMIN‐CTR registration number, UMIN000044130).

### Literature search

Based on a systematic electronic search using PubMed, Web of Science, and the Cochrane Central Register of Controlled Trials (CENTRAL) database, we identified clinical studies published from January 1990 through March 2022, in which treatment outcomes were reported in relation to the timing of interventions for PFCs. The timing of interventions was classified as early (≤4 weeks of the onset of AP) or delayed (>4 weeks) based on the revised Atlanta classification.^3^ Since there were variations between the studies in the thresholds used for the timing of interventions, studies using the threshold of 4 ± 1 weeks were included in the analysis. Two authors (Yousuke Nakai and Hideyuki Shiomi) independently participated in the literature search, study selection, assessment of study quality, and data extraction. Disagreements were resolved through discussion with another author (Tsuyoshi Hamada). The search terms included “pancreatitis”, “pancreatic pseudocyst”, “WON”, “necrotizing pancreatitis”, “drainage”, “treatment”, and “stents”, with their word variations (the search strategy in each database is detailed in Table S1). The search was limited to fully published articles in English and human studies. The search was not limited in terms of patients’ age and length of patient follow‐up. The bibliographies of the identified articles were further screened for additional eligible articles. We included studies involving ≥10 patients per study and excluded studies examining PFCs after pancreatic surgery or trauma, and those reporting treatment outcomes only for surgical management of necrotizing pancreatitis. We also excluded studies when study results of ANC/WON were not separately analyzed from those of acute pancreatic fluid collection and pancreatic pseudocyst.

The quality of reporting data stratified by the timing of interventions for PFCs was assessed using the Newcastle‐Ottawa Scale,^18^ which ranges from 0 (poor quality) to 9 (good quality) summing up the scores for the following three categories: selection of exposed and non‐exposed cohorts (4 points), comparability of cohorts (2 points), and assessment of outcome (3 points). The scores of the included studies are presented in Table S2.

### Data collection

Using a pre‐defined standardized data extraction form, the following data were collected from each study: study design, patient demographics, treatment protocols, treatment outcomes, and outcome definitions. The primary endpoint was adverse events, and secondary endpoints were technical success, clinical success, need for necrosectomy and open surgery, and mortality. The definitions of technical and clinical success were heterogeneous across the studies (Table 1).

**TABLE 1:** Definition of adverse events, technical success, and clinical success

**Study**	**Adverse events**	**Technical success**	**Clinical success**
Guo, 2014^25^	Intra‐abdominal bleeding, and enterocutaneous fistula	NA	NA
Woo, 2017^26^	Defined according to the ASGE lexicon^50^	NA	A reduction in the volume of the necrotic collection to the point where the patient was asymptomatic and was able to be discharged safely
Mallick, 2018^27^	Complications related to drainage like external pancreatic fistula, slippage, blockade of the catheter, and bleeding through the drainage	NA	NA
Trikudanathan, 2018^28^	Defined according to the ASGE lexicon^50^	NA	NA
Oblizajek, 2020^29^	Adverse events likely related to endoscopic intervention	NA	Resolution of the necrotic collection on cross‐sectional imaging after intervention and without surgery
Ganaie, 2021^30^	NA	NA	Recovery with pancreatic cyst drainage alone
Gupta, 2021^31^	NA	NA	NA
Khan, 2021^32^	NA	Successful deployment of the LAMS resulting in drainage of PFC contents into the stomach/duodenal lumen	Resolution of PFC at the time of endoscopic LAMS removal without the requirement for ongoing transmural PFC drainage with DPS or another LAMS
Rana, 2021^33^	Defined according to the ASGE lexicon^50^	Successful placement of EUS‐guided stent (plastic or LAMS) in an initial attempt	Symptomatic improvement accompanied by radiological resolution of PNC and avoidance of surgery
Jagielski, 2022^34^	Gastrointestinal bleeding, stent migration into the lumen of the collection, and gastrointestinal perforation	NA	The lack of collection‐related symptoms and total regression of the collection or collection diameter <40 mm on imaging
Zhang, 2022^35^	Abdominal bleeding, gastrointestinal fistula, symptomatic vein thrombosis	NA	NA

Abbreviations: ASGE, American Society of Gastrointestinal Endoscopy; DPS, double pigtail stent; EUS, endoscopic ultrasonography; LAMS, lumen‐apposing metal stent; NA, not available; PFC, pancreatic fluid collection; PNC, pancreatic necrotic collection.

### Statistical analysis

Using the data reported in the pooled studies, we calculated pooled odds ratios (ORs) and 95% confidence intervals (CIs) for binary outcome variables comparing early to delayed interventions. Given the heterogeneity in study populations and procedures between the studies, we used the DerSimonian‐Laird random‐effects model.^19^ Statistical heterogeneity in outcome variables between the studies was assessed by the Q and *I^2^* statistics.^20^ For the Q statistic, we used a *p*‐value of 0.10 for statistical significance in view of the low power of tests for heterogeneity.^21^ The *I^2^* statistics of around 25%, 50%, and 75% were considered as low‐, moderate‐, and high‐level heterogeneity, respectively.^22^ We assessed potential publication bias by means of the funnel plot with Begg's rank correlation test^23^ and Egger's linear regression test^24^ for assessment of the asymmetry of the funnel plot. A meta‐regression analysis was conducted to assess an association of the proportion of cases receiving a LAMS with pooled OR for an outcome of interest (adverse events [AE] and clinical success).

A two‐sided *p*‐value < 0.05 was considered statistically significant. Given multiple comparisons, the results were interpreted cautiously. All analyses were performed using R software version 4.1.3 and the meta and metatest packages (R Development Core Team, http://www.r‐project.org).

## RESULTS

**FIGURE 1:**
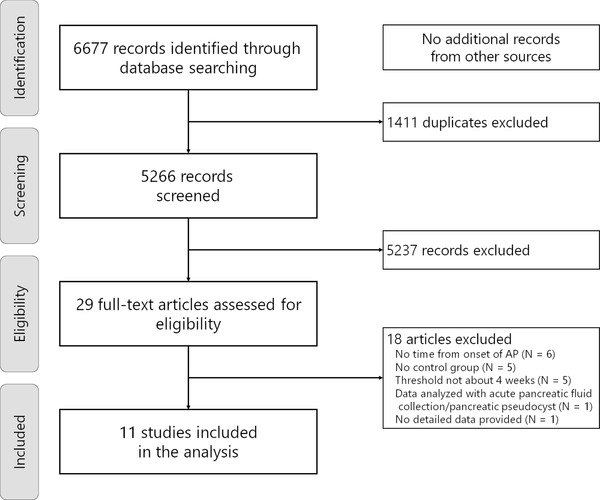
Flowchart of study selection for a meta‐analysis of early and delayed interventions for pancreatic fluid collections

Through the systematic search (Figure 1), we identified 11 eligible studies,^25^, ^26^, ^27^, ^28^, ^29^, ^30^, ^31^, ^32^, ^33^, ^34^, ^35^ involving a total of 1500 patients (775 patients with early interventions and 725 patients with delayed interventions). The characteristics and clinical outcomes of the included studies are summarized in Tables 2 and 3. All studies were conducted based on the retrospective design, and the approach for the initial interventions was endoscopic in four, percutaneous in three, and a combination of endoscopic, percutaneous, or surgical approaches in four. Three studies included an initial surgical approach.^25^, ^26^, ^28^ Patients in the early intervention group were likely to have more organ failure^25^, ^27^, ^35^ as well as less encapsulation^28^, ^29^ and larger collections.^28^, ^29^, ^32^, ^33^, ^34^

**TABLE 2:** Summary of studies included in the meta‐analysis

			**Etiology of pancreatitis**					**Initial approach**			
**Study**	**Group**	***N***	**Alcohol**	**Biliary**	**Organ failure**	**Size of PFC (cm)**	**Complete encapsulation**	**Endoscopic**	**Percutaneous**	**Surgical**	**Follow‐up period**
Guo, 2014^25^	Total	223	24 (11)	108 (48)	82 (37)	NA	NA	0	37 (17)	186 (83)	NA
	Early	136	13 (10)	67 (49)	61 (45)	NA	NA	0	22 (16)	114 (84)	NA
	Delayed	87	11 (13)	41 (47)	21 (24)	NA	NA	0	15 (17)	72 (83)	NA
Woo, 2017^26^	Total	30	3 (10)	13 (43)	NA	NA	NA	12 (40)	8 (27)	10 (33)	NA
	Early	7	NA	NA	NA	NA	NA	NA	NA	NA	NA
	Delayed	23	NA	NA	NA	NA	NA	NA	NA	NA	NA
Mallick, 2018^27^	Total	375	193 (51)	133 (35)	117 (31)	NA	NA	0	375 (100)	0	NA
	Early	258	134 (52)	88 (34)	98 (38)	NA	NA	0	258 (100)	0	NA
	Delayed	117	59 (50)	45 (39)	19 (16)	NA	NA	0	117 (100)	0	NA
Trikudanathan, 2018^28^	Total	193	49 (25)	89 (46)	NA	NA	53 (27)	144 (75)*	45 (23)*	11(6)	NA
	Early	76	19 (25)	34 (45)	NA	17.5 (13.4–23.4)	5 (7)	49 (64)	24 (32)	5 (7)	NA
	Delayed	117	30 (26)	53 (45)	NA	14.0 (9.2–18.6)	48 (43)	95 (81)	21 (18)	6 (5)	NA
Oblizajek, 2020^29^	Total	38	1 (3)	18 (47)	NA	NA	25 (66)	38 (100)	0	0	NA
	Early	19	0	8 (42)	NA	16 (7–24)	8 (42)	19 (100)	0	0	NA
	Delayed	19	1 (5)	10 (53)	NA	15 (5–22)	17 (89)	19 (100)	0	0	NA
Ganaie, 2021^30^	Total	60^†^	10 (17)	30 (50)	8 (13)	NA	NA	0	60 (100)	0	63.2 ± 27 days
	Early	24	NA	NA	NA	NA	NA	0	24 (100)	0	NA
	Delayed	16	NA	NA	NA	NA	NA	0	16 (100)	0	NA
Gupta, 2021^31^	Total	146^‡^	72 (49)	48 (33)	96 (66)	NA	NA	0	144 (100)	0	NA
	Early	90	NA	NA	NA	NA	NA	0	90 (100)	0	NA
	Delayed	54	NA	NA	NA	NA	NA	0	54 (100)	0	NA
Khan, 2021^32^	Total	85	16 (19)	45 (53)	NA	NA	NA	85 (100)	0	0	NA
	Early	6	0	3 (50)	NA	13.0 ± 6.0	NA	6 (100)	0	0	12.8 ± 12.5 weeks
	Delayed	79	16 (21)	42 (56)	NA	11.0 ± 4.4	NA	79 (100)	0	0	15.3 ± 15.2 weeks
Rana, 2021^33^	Total	170	116 (68)	36 (21)	15 (9)	NA	162 (95)	170 (100)	0	0	NA
	Early	34	22 (65)	8 (24)	15 (44)	12.3 ± 2.1	26 (74)	34 (100)	0	0	8.6 ± 4.4 months
	Delayed	136	94 (65)	28 (21)	0	10.5 ± 2.7	136 (100)	136 (100)	0	0	51.4 ± 34.8 months
Jagielski, 2022^34^	Total	71	47 (66)	NA	NA	14.5 ± 6.3	NA	71 (100)	0	0	14 (10‐20) months
	Early	25	20 (80)	NA	NA	18.5 ± 6.8	NA	25 (100)	0	0	NA
	Delayed	46	27 (59)	NA	NA	12.3 ± 4.8	NA	46 (100)	0	0	NA
Zhang, 2022^35^	Total	131	NA	66 (50)	104 (79)§	NA	NA	0	131 (100)	0	NA
	Early	100	NA	51 (51)	83 (83)§	NA	NA	0	100 (100)	0	NA
	Delayed	31	NA	15 (48)	21 (67)§	NA	NA	0	31 (100)	0	NA

*Note*: Numbers are shown in *n* (%), mean ± SD, or median (range).

* A combined endoscopic and percutaneous approach in two in early interventions and five in delayed intervention.

^†^ 40 patients included in the analysis.

^‡^ 144 patients included in the analysis.

^§^Number of patients with multiple organ failure.

Abbreviations: NA, not available; PFC, pancreatic fluid collection.

**TABLE 3:** Clinical outcomes of early and delayed interventions for pancreatic fluid collections

**Study**	**Group**	***N***	**New organ failure**	**ICU stay**	**Length of hospital stay (days)**	**Time to resolution (days)**
Guo, 2014^25^	Early	136	20 (15)	NA	NA	NA
	Delayed	87	7 (8)	NA	NA	NA
Woo, 2017^26^	Early	7	NA	NA	137 (NA)	NA
	Delayed	23	NA	NA	NA	NA
Mallick, 2018^27^	Early	258	NA	NA	22.0 ± 13.6	28.4 ± 20.7
	Delayed	117	NA	NA	22.9 ± 12.6	30.2 ± 26.2
Trikudanathan, 2018^28^	Early	76	NA	2.5 (0–22)^*^, ^†^	37 (IQR 27–61)^*^, ^†^	NA
	Delayed	117	NA	0 (0–3)^*^, ^†^	26 (IQR 0–207)^*^, ^†^	NA
Oblizajek, 2020^29^	Early	19	NA	1 (0–22)^†^	26 (6–44)^†^	103 (44–422)^†^
	Delayed	19	NA	0 (0–2)^†^	6 (0–40)^†^	69 (27–330)^†^
Ganaie, 2021^30^	Early	24	NA	NA	NA	NA
	Delayed	16	NA	NA	NA	NA
Gupta, 2021^31^	Early	90	NA	NA	NA	NA
	Delayed	54	NA	NA	NA	NA
Khan, 2021^32^	Early	6	NA	NA	NA	56.5 ± 28.5
	Delayed	79	NA	NA	NA	46.3 ± 35.6
Rana, 2021^33^	Early	34	NA	NA	NA	31.6 ± 6.0
	Delayed	136	NA	NA	NA	29.5 ± 8.5
Jagielski, 2022^34^	Early	25	NA	NA	NA	NA
	Delayed	46	NA	NA	NA	NA
Zhang, 2022^35^	Early	100	8 (8)^‡^	30.0 (17.0–48.0)	42.5 (24.3–68.5)	NA
	Delayed	31	2 (6)^‡^	22.0 (9.0–55.0)	40.0 (24.0–71.0)	NA

Numbers are shown in *n* (%), mean ± SD, or median (range), unless otherwise indicated.

* median (interquartile range).

^†^ *p* < 0.05 for a comparison between early and delayed interventions.

^‡^ Number of patients with new multiple organ failure (this means Guo's study reports the rate of cases with organ failure including those presenting with organ failure).

Abbreviations: ICU, intensive care unit; IQR, interquartile range; NA, not available; PFC, pancreatic fluid collection.

**FIGURE 2:**
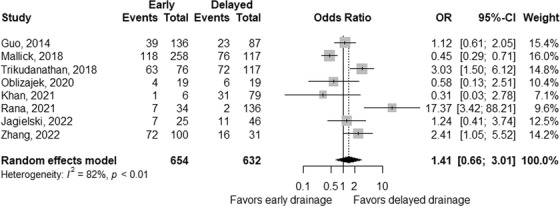
Comparison of adverse events between early and delayed interventions. The odds ratio (OR) for early intervention compared with delayed intervention is presented for each study (center of the gray square) with a 95% confidence interval (CI; horizontal line). Summary OR based on a meta‐analysis via the random‐effect model is presented at the bottom (center of the black diamond) with 95% CI (the width of the black diamond). The *p*‐value for the Q‐statistic for between‐study heterogeneity is shown

**FIGURE 3:**
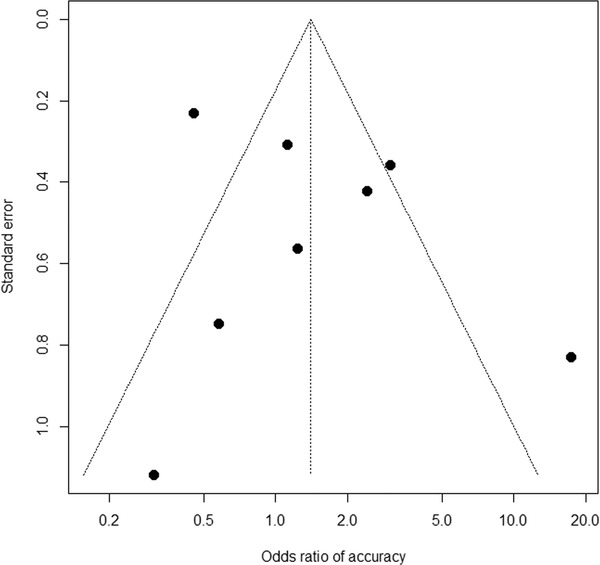
Funnel plots of adverse events to examine potential publication bias in odds ratio. Each dot indicates a respective study. Diagonal dotted lines indicate 95% confidence limits. *p* = 0.80 for Begg's rank correlation test and *p* = 0.32 for Egger's linear regression test

The summary of pooled ORs according to the treatment approach is shown in Table 4. Based on eight studies,^25^, ^27^, ^28^, ^29^, ^32^, ^33^, ^34^, ^35^ the rate of adverse events was comparable, with a pooled OR of 1.41 (95% CI 0.66–3.01; *p* = 0.38; Figure 2) for early interventions compared to delayed interventions, though the data were heterogeneous between the studies (*p*_heterogeneity_ < 0.01 and *I^2^* = 82%). Based on quantitative measurement using Egger's test as well as visual inspection of the funnel plot, there was no significant evidence of publication bias in reporting adverse events (Figure 3). The rates of bleeding^25^, ^27^, ^28^, ^29^, ^32^, ^33^, ^34^, ^35^ showed a similar tendency, with a pooled OR of 1.35 (95% CI 0.72–2.53; *p* = 0.36) and potential heterogeneity between the studies (*p*_heterogeneity_ = 0.05 and *I^2^* = 49%). The results were consistent when three studies including the initial surgical approach were excluded from the analysis (Table 4). When adverse events of studies either by endoscopic or percutaneous approach alone were analyzed, pooled ORs were 1.47 (95% CI 0.28–7.79; *p* = 0.65) in four studies including only endoscopic approach,^8^, ^29^, ^32^, ^33^, ^34^ and 1.01 (95% CI 0.20–5.16; *p* = 0.99) in 2 studies including only percutaneous approach.^27^, ^35^

**TABLE 4:** Pooled odds ratio according to treatment approaches

	**All studies (*n* = 11)**			**Studies on non‐surgical treatment (*n* = 8)**			**Studies on endoscopic treatment (*n* = 4)**			**Studies on percutaneous treatment (*n* = 3)**		
**Outcome**	**No. of studies**	**Pooled OR (95% CI)**	***p***	**No. of studies**	**Pooled OR (95% CI)**	***p***	**No. of studies**	**Pooled OR (95% CI)**	***p***	**No. of studies**	**Pooled OR (95% CI)**	***p***
Adverse events	8	1.41 (0.66–3.01)	0.38	6	1.27 (0.43–3.73)	0.66	4	1.47 (0.28–7.79)	0.65	2	1.01 (0.20–5.16)	0.99
Bleeding	8	1.35 (0.72–2.53)	0.36	6	1.46 (0.49–4.30)	0.49	4	2.17 (0.37–12.6)	0.39	2	0.91 (0.47–1.76)	0.79
Mortality	11	1.70 (1.21–2.40)	<0.01	8	1.49 (0.99–2.24)	0.06	4	3.04 (0.57–16.1)	0.19	3	1.36 (0.83–2.22)	0.23
Clinical success	5	0.39 (0.15–1.00)	0.05	5	0.39 (0.15–1.00)	0.05	4	0.45 (0.13–1.61)	0.22	1	0.33 (0.08–1.33)	0.12
Requirement of necrosectomy	7	2.14 (0.83–5.54)	0.11	6	2.23 (0.71–6.99)	0.17	4	2.72 (0.44–16.7)	0.28	2	1.39 (0.82–2.36)	0.23
Requirement of surgery	9	1.23 (0.64–2.37)	0.54	7	1.28 (0.83–1.96)	0.26	3	5.62 (0.91–34.6)	0.06	3	1.05 (0.64–1.75)	0.84

Abbreviations: CI, confidence interval; OR, odds ratio.

**FIGURE 4:**
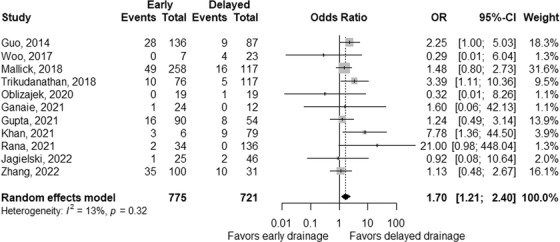
Comparison of mortality between early and delayed interventions. The odds ratio (OR) for early intervention compared with delayed intervention is presented for each study (center of the gray square) with a 95% confidence interval (CI; horizontal line). Summary OR based on a meta‐analysis via the random‐effect model is presented at the bottom (center of the black diamond) with 95% CI (the width of the black diamond). The *p*‐value for the Q‐statistic for between‐study heterogeneity is shown

The rate of mortality was significantly higher in early interventions, with a pooled OR of 1.70 (95% CI 1.21–2.40, *p* < 0.01; Figure 4), without significant heterogeneity between studies (*p*_heterogeneity_ = 0.32 and *I^2^* = 13%).^25^, ^26^, ^27^, ^28^, ^29^, ^30^, ^31^, ^32^, ^33^, ^34^, ^35^ Pooled ORs were 1.49 (95% CI 0.99–2.24, *p* = 0.06; Figure S1a) in 8 studies without surgical approach, 3.04 (95% CI 0.57–16.05, *p* = 0.19; Figure S1b) in four studies with endoscopic approach^29^, ^32^, ^33^, ^34^ and 1.36 (95% CI 0.83–2.22, *p* = 0.23, Figure S1c) in three studies with percutaneous approach.^27^, ^30^, ^35^

**FIGURE 5:**
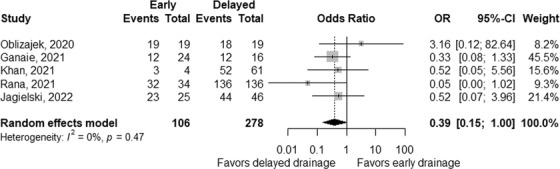
Comparison of clinical success between early and delayed interventions. The odds ratio (OR) for early intervention compared with delayed intervention is presented for each study (center of the gray square) with a 95% confidence interval (CI; horizontal line). Summary OR based on a meta‐analysis via the random‐effect model is presented at the bottom (center of the black diamond) with 95% CI (the width of the black diamond). The *p*‐value for the Q‐statistic for between‐study heterogeneity is shown

Technical success rates were reported in two studies; 100% in both groups in one study,^32^, ^33^ and 100% and 95% in early and delayed interventions in the other study.^32^ Based on five studies,^29^, ^30^, ^32^, ^33^, ^34^ which did not include initial surgical interventions, clinical success rates tended to be low in early interventions with a pooled OR of 0.39 (95% CI 0.15–1.00; *p* = 0.05; Figure 5), without significant heterogeneity between the studies (*p*_heterogeneity_ = 0.47 and *I^2^* = 0%).

The rates of necrosectomy tended to be high in early interventions, with a pooled OR of 2.14 (95% CI 0.83–5.54; *p* = 0.11; Figure S2), but the data were heterogeneous between the studies (*p*_heterogeneity_ < 0.01 and *I^2^* = 81%).^8^, ^27^, ^28^, ^29^, ^32^, ^33^, ^34^, ^35^ Meanwhile, a pooled OR of open surgery was 1.23 (95% CI 0.64–2.37; *p* = 0.54; Figure S3), but again with significant heterogeneity (*p*_heterogeneity_ < 0.01 and *I^2^* = 60%).^8^, ^25^, ^27^, ^28^, ^29^, ^30^, ^31^, ^32^, ^33^, ^35^

Other clinical outcomes are shown in Table 3. Two studies^28^, ^29^ revealed intensive care unit and hospital stay was significantly longer in early interventions. New organ failure was reported in two studies^25^, ^35^ but the rates did not differ by the timing of interventions.

Among four studies that evaluated exclusively the initial endoscopic interventions,^29^, ^32^, ^33^, ^34^ the rates of LAMS use ranged between 14% and 100%. Meta‐regression was performed for AE and clinical success according to the proportion of LAMSs used in each study. The use of LAMS did not correlate with AE (regression coefficient, −0.02; 95% CI −0.24–0.19; *p* = 0.82) or clinical success (regression coefficient, 0.08; 95% CI −0.16–0.32; *p* = 0.50).

## DISCUSSION

In this meta‐analysis, we investigated the impact of timing of intervention from the onset of AP on clinical outcomes of necrotizing pancreatitis and showed that the adverse event rate was comparable but the mortality rate was significantly higher in early interventions. In addition, the clinical success rate did not improve in early interventions. Our findings suggest that delayed interventions, followed by the step‐up approach, which is usually selected in the current clinical practice, are reasonable in terms of safety and effectiveness.

There are two major advantages to the delayed approach. First, some necrotizing pancreatitis can resolve without intervention and the delayed approach can avoid unnecessary interventions, which are potentially associated with adverse events. About 60% of necrotizing pancreatitis^36^ and 40% of infected necrotizing pancreatitis^37^ resolve by conservative treatment alone. The second advantage of the delayed approach is the complete encapsulation of ANCs, which can reduce the risk of spilling infected necrotic tissue and thereby, allow safe interventions, including necrosectomy, and ANCs are often walled‐off after 3–4 weeks, as described in the revised Atlanta classification.^3^ Some ANCs were encapsulated within 4 weeks of the onset of AP, but the reported rates of complete encapsulation were equal to or higher in delayed interventions (Table 2). These data may implicate that the differences in the degree of encapsulation per se cannot explain our findings of a higher mortality rate in the early drainage group. In some cases with early interventions, clinical symptoms might be caused by the ongoing process of AP, not infections of ANCs, and interventions such as drainage and necrosectomy would not improve clinical outcomes in this situation. However, differentiation between those two conditions can be difficult in clinical practice and there is often a dilemma whether we should intervene early in cases with the deteriorated condition, or rather wait for complete encapsulation.

Meanwhile, proactive drainage for necrotizing pancreatitis even without clinical signs of infection is increasingly reported because infection does occur early in the course of necrotizing pancreatitis.^11^ The established strategy of less invasive non‐surgical management of PFCs including the wide spread of LAMS^38^, ^39^, ^40^ has dramatically accelerated this concept of early proactive interventions. However, our meta‐analysis did not provide evidence supporting routine interventions at an early stage of PFCs in terms of safety and effectiveness. Nonetheless, it should be noted that including only retrospective studies in the current study and resultant between‐group imbalance in patient profiles might result in apparently high rates of morbidity and mortality in the early intervention group, that is, more common organ failure in early interventions was likely to affect clinical outcomes negatively.

Development of new organ failure following intervention for necrotizing pancreatitis, as well as organ failure at presentation,^41^ may worsen clinical outcomes of necrotizing pancreatitis. Early interventions during the acute inflammatory phase may increase the risk of systemic inflammatory response syndrome and new‐onset organ failure, whereas deteriorated infections by delayed interventions may also increase the risk of systemic inflammatory response syndrome.^42^ In our meta‐analysis, the rates of new‐onset organ failure did not differ by the timing of interventions (Table 3), despite the limited number of studies reporting the corresponding data.^25^, ^27^, ^35^ Early interventions may potentially enhance the resolution of infection and shorten hospital stay, but our data suggest that the clinical success rate was non‐significantly lower and the length of hospital or intensive care unit stay was not shortened in early interventions. Historically, early surgical interventions for necrotizing pancreatitis did not necessarily lead to better clinical outcomes, and this might be also true for the less invasive endoscopic and percutaneous approaches. Thus, as discussed above, to maximize the potential benefits of early interventions, we should select cases with symptomatic ANCs due to infection, not ongoing AP, and further exploration of biomarkers for infected ANCs is warranted.^43^

A recent randomized controlled trial of immediate or postponed drainage for infected necrotizing pancreatitis^37^ needs comments. Randomization was performed at the time of diagnosis of infected necrosis, not at 4 weeks from the onset of AP, but this randomized controlled trial failed to demonstrate the superiority of immediate drainage at the diagnosis of infected necrosis in terms of complications and mortality as compared to postponed interventions, in line with our meta‐analysis. In a retrospective study by the same Dutch Pancreatitis Study group,^44^ the researchers demonstrated less need for necrosectomy and reduced in‐hospital mortality by the early proactive approach. The percutaneous approach utilized in these two studies^44^, ^45^ is theoretically more sterile than the endoscopic approach and can be safe even in early interventions for ANCs without encapsulation.^46^ However, morbidity and mortality of early interventions were not low in the percutaneous approach in our meta‐analysis. While LAMS allows better drainage through its large bore, its deployment needs some techniques in less liquified ANCs.^47^, ^48^ Thus, it is still unclear whether the use of LAMS as early drainage is safe and effective in necrotizing pancreatitis. In our meta‐regression analysis, the proportion of LAMS use did not correlate with the rate of AE or clinical success but it should be further evaluated whether endoscopic drainage by large bore LAMS rather than percutaneous drainage would increase treatment safety and efficacy in this setting. Of note, a similar meta‐analysis including one randomized controlled trial by Boxhoorn et al.^37^ was recently reported by Gao et al.,^49^ but the definition of early interventions was various including the timing from hospitalization, not the onset of AP. Furthermore, we were able to include some additional studies by meticulous evaluation of studies eligible for the meta‐analysis.

There are some limitations to the current meta‐analysis. First, the risk of bias is high since the number of cases was limited and only retrospective studies were included in the analysis. Due to the retrospective designs of the included studies, the risk of bias was present in our meta‐analysis. In clinical practice, for example, we tend to intervene early in cases with deteriorating conditions, which can lead to higher mortality in the early intervention group. In addition, our meta‐analysis was also limited by heterogeneity among studies. For example, *p*_heterogeneity_ was < 0.01 in our primary endpoint of adverse events. The follow‐up period was available only in four studies and varied widely, too. The differences in the follow‐up period might affect some clinical outcomes in our meta‐analysis. Secondly, although we only included the studies using the threshold of 4 ±1 weeks from the onset of AP, the onset of AP is not always clear. Given the concept of waiting until the “walled‐off” approach, the data on the status of encapsulation might be rather important than the timing of intervention. Chantarojanasiri et al.^8^ did include encapsulation of PFC on computed tomography as the indication of endoscopic ultrasound‐guided drainage.

In conclusion, our meta‐analysis did not support routine utilization of early interventions in necrotizing pancreatitis since the mortality rate is higher without improvement in clinical success. However, further studies are warranted on whether early interventions may have a role in some subgroups, such as encapsulated ANCs and ANCs without ongoing AP.

## CONFLICT OF INTEREST

Yousuke Nakai received research grants from Boston Scientific Japan, HOYA Corporation, and honoraria from Boston Scientific Japan, Fujifilm Corporation, and Olympus Corporation. Hiroyuki Isayama received research grants from Boston Scientific Japan, Fujifilm Corporation, Fujifilm Health Care Corporation, Gaderius Medical KK, Zeon Medical Inc., and honoraria from Boston Scientific Japan, Fujifilm Corporation, Taewoong Medical Devices, Olympus Corporation, Century Medical, Inc. and Cook Medical Japan G.K.

Ichiro Yasuda is an associate editor of DEN Open, and Yousuke Nakai, Mamoru Takenaka, and Takuji Iwashita are associate editors of Digestive Endoscopy.

## FUNDING INFORMATION

None.

## Supporting information




**Figure S1a**: Comparison of mortality between early and delayed interventions by non‐surgical approach.

**Figure S1b**: Comparison of mortality between early and delayed interventions by endoscopic approach.

**Figure S1c**: Comparison of mortality between early and delayed interventions by percutaneous approach.

**Figure S2**: Comparison of necrosectomy rate between early and delayed interventions.

**Figure S3**: Comparison of open surgery rate between early and delayed interventions.

**Table S1**: Strategies of database search for studies reporting clinical outcomes of endoscopic ultrasound‐guided treatment of pancreatic fluid collections
**Table S2**: The Newcastle‐Ottawa Scale for assessment of data reporting quality of each study included in the meta‐analysis
