# Supplementary figures and images for: Early versus delayed interventions for necrotizing pancreatitis: A systematic review and meta‐analysis
Source: DEN Open. 2022 Oct 10;3(1):e171. doi: 10.1002/deo2.171 (PMC9549879; doi:10.1002/deo2.171)

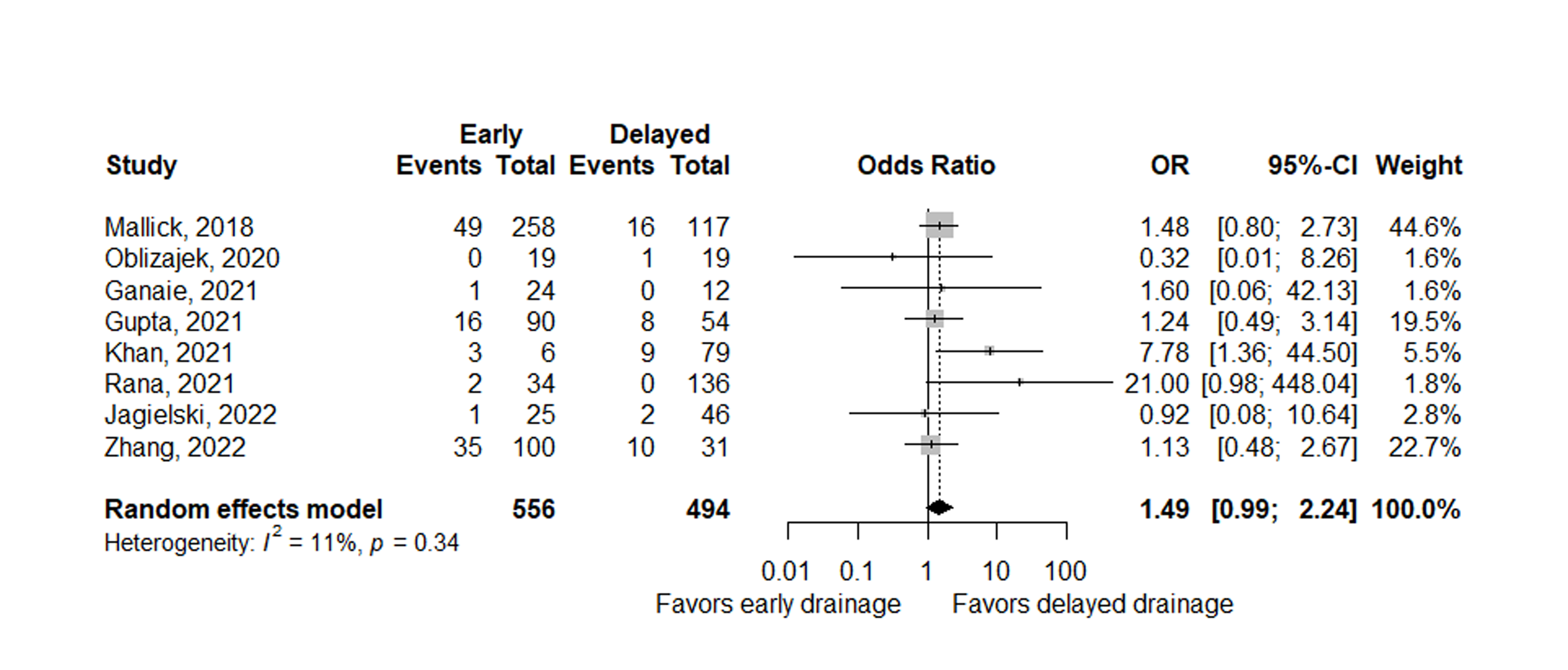

Supplement: Supplementary file 1 — Figure S1a: Comparison of mortality between early and delayed interventions by non‐surgical approach. [file DEO2-3-e171-s001.tif]

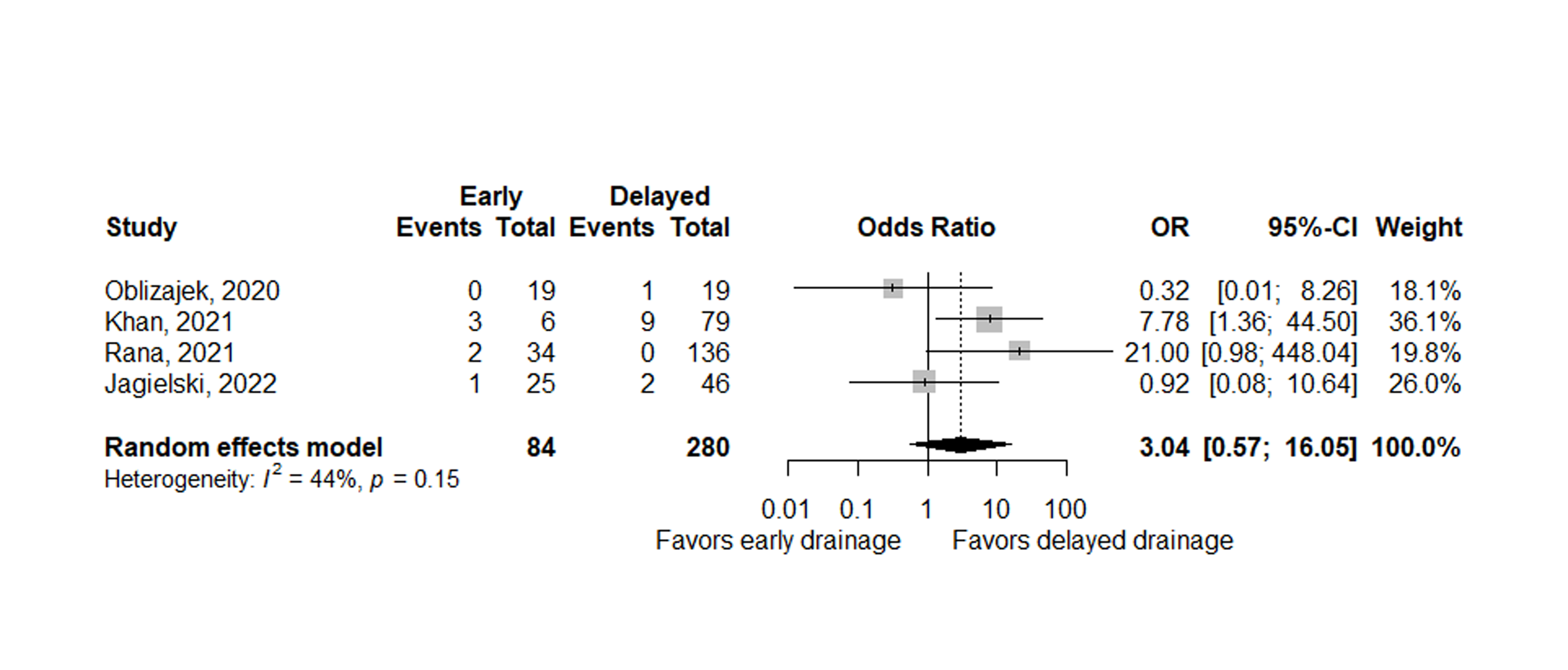

Supplement: Supplementary file 2 — Figure S1b: Comparison of mortality between early and delayed interventions by endoscopic approach. [file DEO2-3-e171-s004.tif]

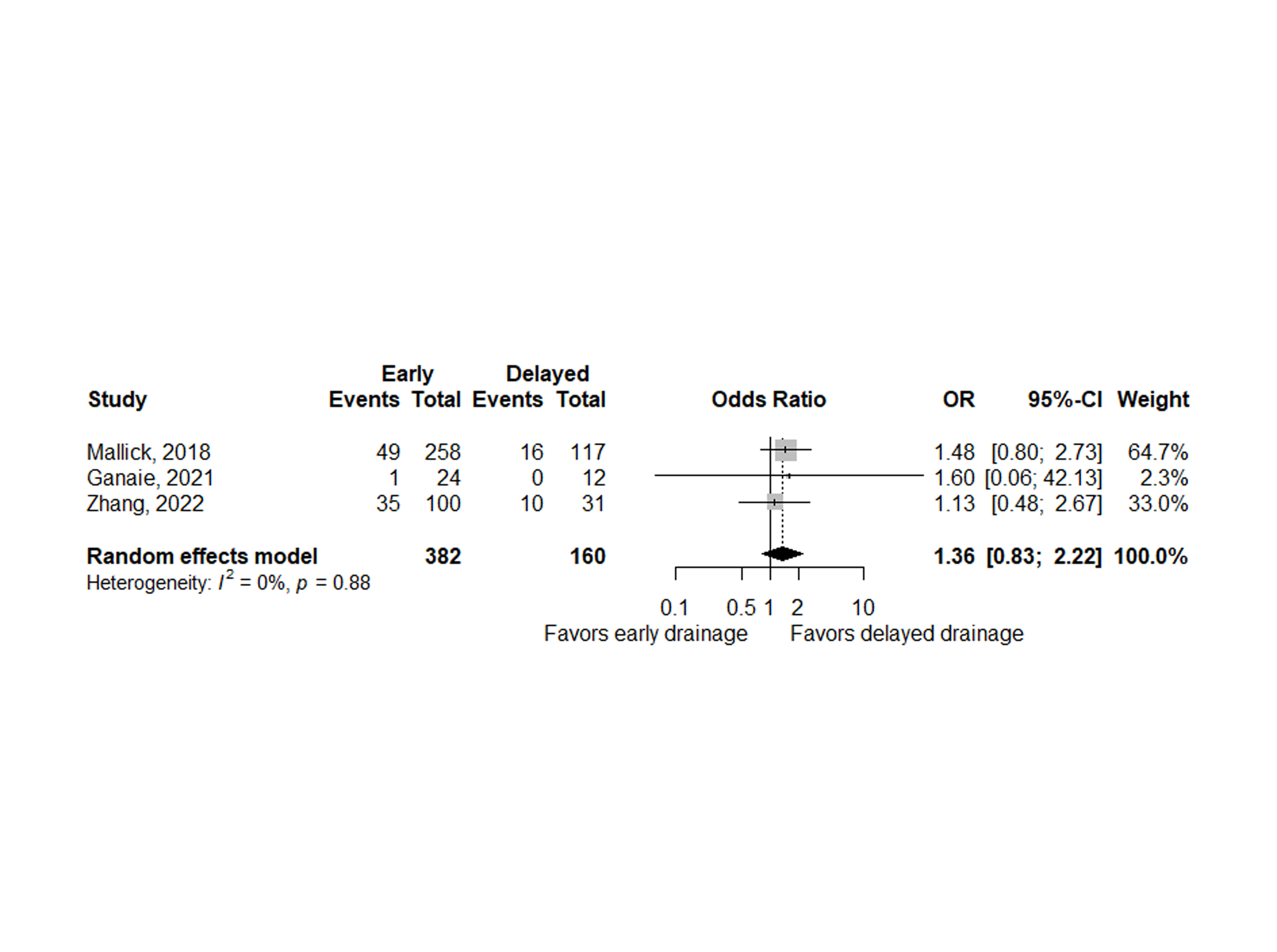

Supplement: Supplementary file 3 — Figure S1c: Comparison of mortality between early and delayed interventions by percutaneous approach. [file DEO2-3-e171-s006.tif]

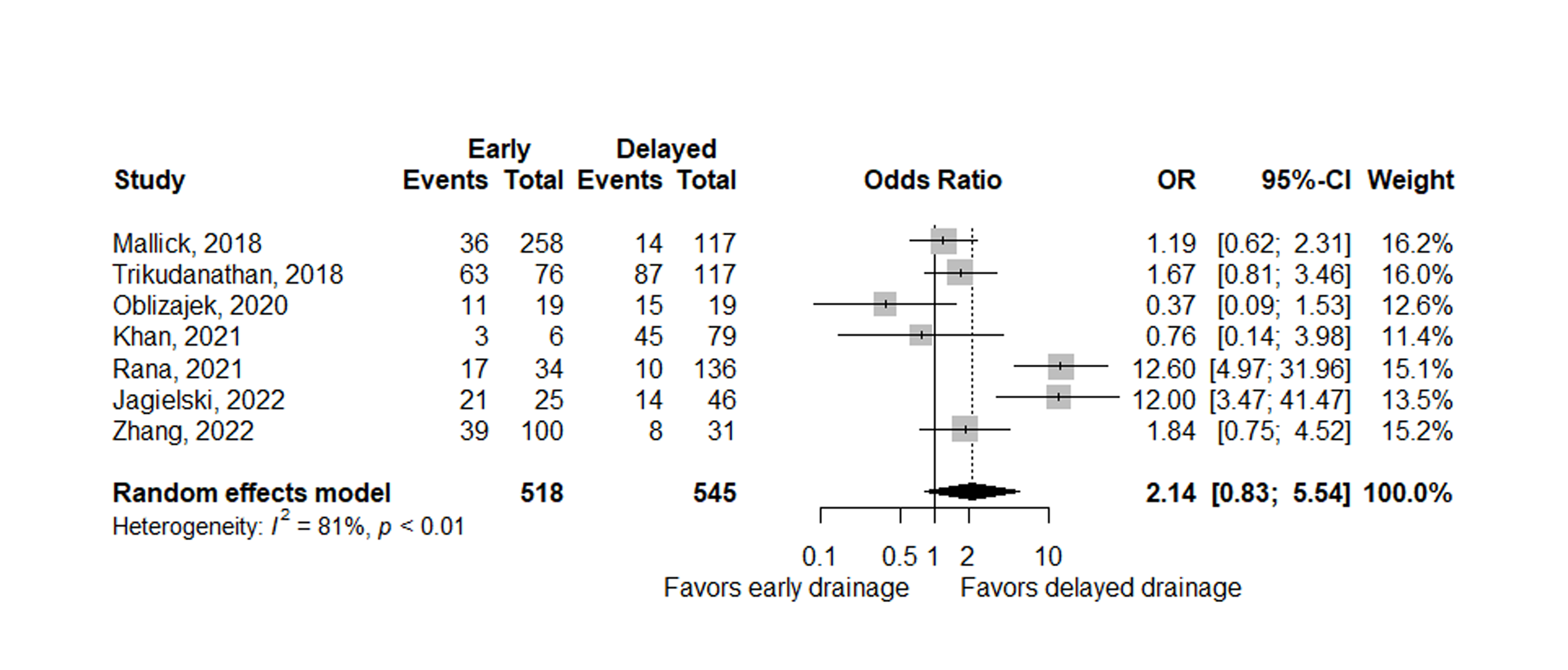

Supplement: Supplementary file 4 — Figure S2: Comparison of necrosectomy rate between early and delayed interventions. [file DEO2-3-e171-s005.tif]

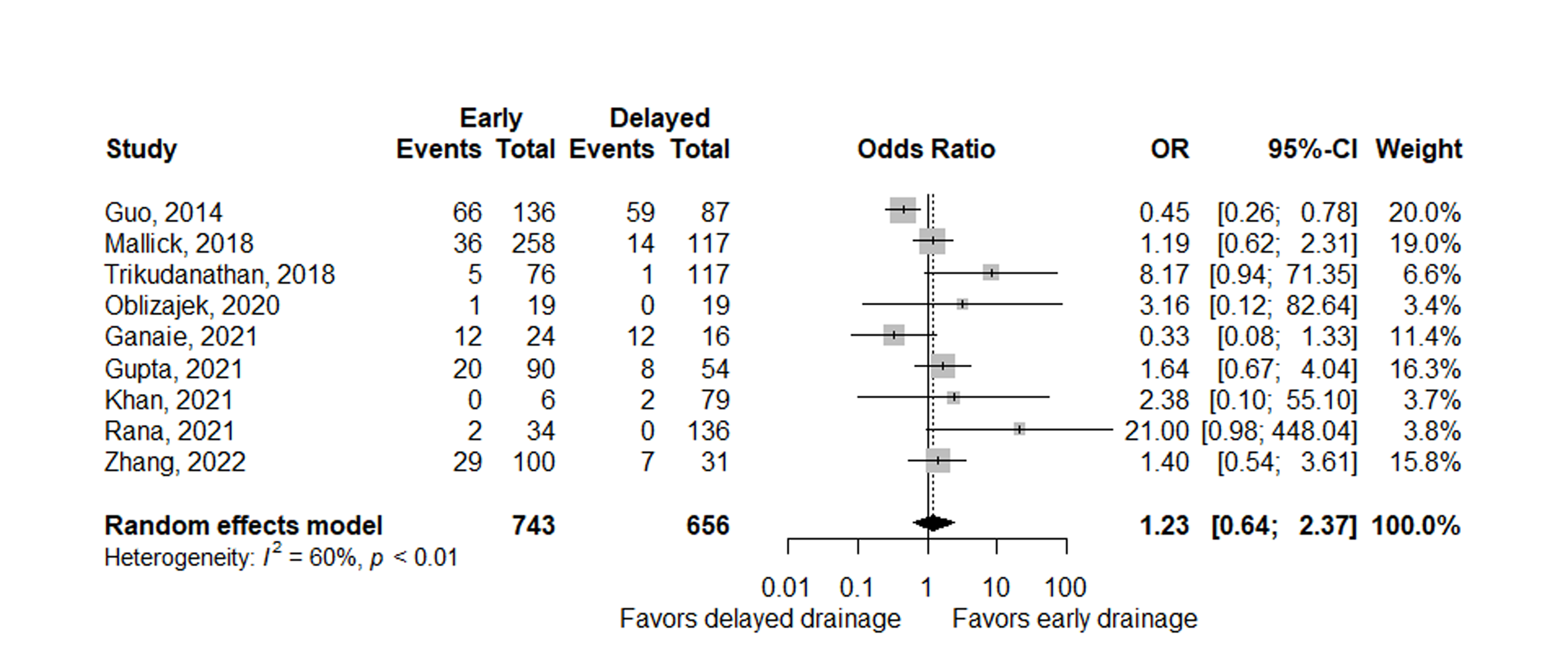

Supplement: Supplementary file 5 — Figure S3: Comparison of open surgery rate between early and delayed interventions. [file DEO2-3-e171-s003.tif]
