# Supplementary material for: Early versus delayed interventions for necrotizing pancreatitis: A systematic review and meta‐analysis
Source: DEN Open. 2022 Oct 10;3(1):e171. doi: 10.1002/deo2.171 (PMC9549879; doi:10.1002/deo2.171)
Supplement: Supplementary file 6 — Table S1: Strategies of database search for studies reporting clinical outcomes of endoscopic ultrasound‐guided treatment of pancreatic fluid collections Table S2: The Newcastle‐Ottawa Scale for assessment of data reporting quality of each study included in the meta‐analysis [file DEO2-3-e171-s002.docx]

Supplementary Table 1. Strategies of database search for studies reporting clinical outcomes of endoscopic ultrasound-guided treatment of pancreatic fluid collections

| Database | Search terms and limitations |
| --- | --- |
| PubMed  (*n* = 1935) | Searched on 4/1/2022  (pancreatic fluid collection [Title/Abstract] OR pancreatic pseudocyst [Title/Abstract] OR walled-off necrosis [Title/Abstract] OR necrotizing pancreatitis [Title/Abstract]) AND (drainage [Title/Abstract] OR stent [Title/Abstract] OR treatment [Title/Abstract]) AND (“1990/1/1” [Date - Publication] : “2022/3/31” [Date - Publication]) AND (English [Language]) |
|  |  |
| Web of Science  (*n* = 4610) | Searched on 4/1/2022  (TI=((pancreatic fluid collection) OR (pancreatic pseudocyst) OR (walled-off necrosis) OR (necrotizing pancreatitis)) OR AB = ((pancreatic fluid collection) OR (pancreatic pseudocyst) OR (walled-off necrosis) OR (necrotizing pancreatitis))) AND (TI=((drainage) OR (stent) OR (treatment)) OR AB=((drainage) OR (stent) OR (treatment)))  Limitations: “published date between 1999/1/1 and 2022/3/31” |
|  |  |
| Cochrane Library (*n* = 132) | Searched on 4/1/2022  (pancreatic fluid collection or pancreatic pseudocyst or walled-off necrosis or necrotizing pancreatitis) and (drainage or stent or treatment)  Word variations were searched.  Limitations: “title, abstract”, “English Lungage”, and “published between 1990 and 2022” |
|  |  |

Supplementary Table 2. The Newcastle-Ottawa Scale for assessment of data reporting quality of each study included in the meta-analysis

|  | Selection | Comparability | Exposure |
| --- | --- | --- | --- |
| Guo, 2014 | ** | * | ** |
| Woo, 2017 | ** | * | ** |
| Mallick, 2018 | ** | * | ** |
| Trikudanathan, 2018 | ** | * | ** |
| Oblizajek, 2020 | ** | ** | ** |
| Ganaie, 2021 | ** | * | ** |
| Gupta, 2021 | * | * | ** |
| Khan, 2021 | ** | * | ** |
| Rana, 2021 | ** | * | ** |
| Jagielski, 2022 | ** | * | ** |
| Zhang, 2022 | ** | * | ** |
| Study can be awarded a maximum of 4 stars for Selection 2 stars for Comparability, and 3 stars for Exposure. | | | |

Study can be awarded a maximum of 4 stars for Selection, 2 stars for Comparability, and 3 stars for Exposure. According to the total score (the number of stars), the quality of data reporting was categorized as poor (0-2 points), fair (3-6 points), and good (≥7 points).
